# The combined effects of overweight/obesity and dietary antioxidant quality score on hypertension in children and adolescents

**DOI:** 10.1186/s12887-023-04397-0

**Published:** 2023-11-21

**Authors:** Ying Zhong, Zhiqun Zhang, Youfang Hu

**Affiliations:** 1 Department of Children’s Health Care, The First Affiliated Hospital of Nanjing Medical University, Nanjing, Jiangsu 210036 China; 2 Department of Neonatology, Affiliated Hangzhou First People’s Hospital, School of Medicine, Westlake University, Hangzhou, Zhejiang 310000 China

**Keywords:** Obesity, Dietary antioxidant quality score, Hypertension, Children and adolescents

## Abstract

**Background:**

This study was to evaluate the combined effects of overweight/obesity and DAQS on the risk of hypertension in children and adolescents.

**Methods:**

In this cross-sectional study, the data of 14,316 subjects were extracted from the National Health and Nutrition Examination Survey (NHANES). Multivariate logistic regression analysis was used to explore the associations of obesity and DAQS with the risk of hypertension. The combined effect of overweight/obesity and DAQS on the risk of hypertension was evaluated.

**Results:**

Body mass index (BMI)-for-age < 85^th^ percentile was associated with reduced risk of hypertension in children and adolescents [odds ratio (OR) = 0.48, 95% confidence interval (CI): 0.41–0.62]. No significant association between DAQS ≥ 3 and the risk of hypertension before and after the adjustment of confounders (*P* > 0.05). Subjects with BMI-for-age of < 85^th^ percentile and DAQS < 3 was associated with decreased risk of hypertension (OR = 0.53, 95%CI: 0.35–0.79). People with BMI-for-age of < 85^th^ percentile and DAQS ≥ 3 was correlated with decreased risk of hypertension (OR = 0.52, 95%CI: 0.36–0.74). Subgroup analysis revealed that in subjects aged ≥ 12 years, decreased risk of hypertension was observed in BMI-for-age < 85^th^ percentile and DAQS < 3 group (OR = 0.48, 95%CI: 0.31–0.73) as well as BMI-for-age < 85^th^ percentile and DAQS ≥ 3 group (OR = 0.47, 95%CI: 0.32–0.67). In boys, BMI-for-age < 85^th^ percentile and DAQS < 3 group (OR = 0.45, 95%CI: 0.25–0.81) as well as BMI-for-age < 85^th^ percentile and DAQS ≥ 3 group (OR = 0.40, 95%CI: 0.25–0.65) were correlated with decreased risk of hypertension.

**Conclusion:**

Overweight/obesity and DAQS had combined effects on the risk of hypertension in children and adolescents, which implied that for children and adolescents with normal weight, to keep normal weight combined with high quality of diet might be recommended.

## Background

High blood pressure and hypertension in children and adolescents are growing health problems frequently overlooked [1]. Increasing evidence indicated that the prevalence of elevated blood pressure and hypertension in children and adolescents was significantly increased over the past 20 years, which was higher than it was thought before [2, 3]. A previous study estimated that there were 3.5%-5% of children and adolescents suffered from hypertension [4]. Children and adolescents with hypertension are at an increased risk for hypertension in early adulthood, and for early subclinical cardiovascular morbidity [5]. To explore more factors associated with the risk of hypertension in children and adolescents is essential for the management of this disease.

Previous evidence showed that oxidative stress and chronic inflammation play important roles in the pathological mechanism of hypertension in children and adolescents through a variety of factors affecting the occurrence of hypertension [6]. Overweight and obesity are factors promoting oxidative stress and inflammation, and various studies confirmed the association between childhood obesity and current and adult hypertension [7–10]. On the other hand, dietary antioxidant nutrients were reported to be associated with the risk of hypertension [11, 12], but the evidence in children and adolescents is limited. In addition, a single antioxidant nutrient has certain limitations in evaluating the total dietary antioxidant capacity, and dietary antioxidant quality score (DAQS) based on multiple antioxidant nutrients including vitamin A, vitamin E, vitamin C, zinc, selenium and magnesium might be more comprehensively reflecting the dietary antioxidant capacity, which has been applied in studies on overweight and obesity in children [13, 14]. The association of DAQS with hypertension in children and adolescents was still unclear. There was also a study showing a positive correlation between DAQS and waist circumference and waist-to-hip ratio in children with emaciation and normal weight [14]. Another study revealed that dietary antioxidant level was negatively correlated with body mass index (BMI) and total fat in obese children [15]. Thus, we suspected that BMI and DAQS might have combined effects on hypertension in children and adolescents.

This study aimed to explore the influence and combined effects of overweight/obesity and DAQS on the risk of hypertension in children and adolescents based on the data from the National Health and Nutrition Examination Survey (NHANES).

## Methods

### Study design and population

**Fig. 1:**
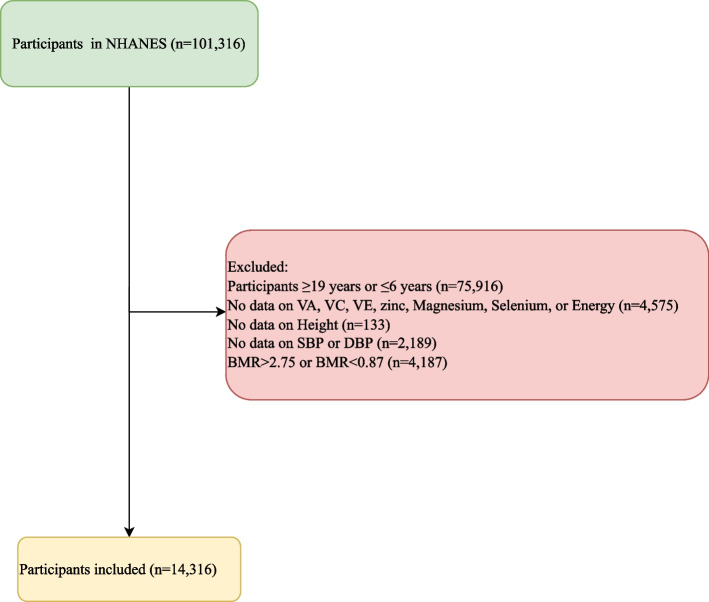
The screen process of the participants

In this cross-sectional study, the data of 101,316 participants between 1999 and 2018 were extracted from the NHANES. The NHANES is a research project of the National Center for Health Statistics (NCHS) collecting the health and nutrition statistics of the United States population and to ensure that the study participants are representative, the organization implemented a stratified, multistage, and clustered probability sampling design [16]. The inclusion criteria were as follows: 1) aged 6–19 years old, 2) completed at least 1 valid 24-h dietary recall and 3) who could be assessed for blood pressure or had been diagnosed for hypertension or taken prescription for hypertension. Subjects who aged ≥ 19 years or ≤ 6 years (*n* = 75,916) were excluded, participants without data on vitamin A, vitamin E, vitamin C, zinc, magnesium, selenium, or energy (*n* = 4,575), people without data on height (*n* = 133), SBP or DBP (*n* = 2,189), and those with misreporting of energy intake (*n* = 4,187) were also excluded. Finally, 14,316 participants were analyzed. The detailed screen process was shown in Fig. 1. The requirement of ethical approval for this was waived by the Institutional Review Board of The First Affiliated Hospital of Nanjing Medical University, because the data was accessed from NHANES (a publicly available database). Written informed consent from the participants’ legal guardian/next of kin was waived by the Institutional Review Board of The First Affiliated Hospital of Nanjing Medical University due to retrospective nature of the study. All methods were performed in accordance with the relevant guidelines and regulations.

### Potential covariates

Age (year), gender (male or female), race (Mexican American, other Hispanic, non-Hispanic White, non-Hispanic Black, or other race including multi-racial), poverty income ratio (PIR), does anyone smoke in the home (no or yes), weight at birth (< 5.5 pounds, 5.5 pounds -9 pounds, or ≥ 9 pounds), physical activity (≤ 3 days, 3–4 days or ≥ 5 days), and energy (kcal).

### Main and outcome variables

BMI-for-age and DAQS were main variables in our study. Overweight and obesity were defined based on BMI-for-age weight status categories, and overweight referred to those with 85^th^ to less than the 95^th^ percentile while obesity referred to those ≥ the 95^th^ percentile [17].

The calculation of DAQS was based on six nutrients, namely vitamin A, vitamin C, vitamin E, zinc, magnesium, and selenium. These dietary antioxidant micronutrients data were collected following the MEC visit and conducted over the telephone. The dietary intake component of NHANES, called What We Eat in America, is conducted as a partnership between the US Department of Agriculture (USDA) and the US Department of Health and Human Services. Under this partnership, NHANES uses the USDA’s Automated Multiple Pass Method (AMPM) to collect 24-h dietary recalls by trained dietary interviewers, which was utilized to estimate the types and quantities of food groups and nutrients consumed. The initial dietary recall interview was conducted face-to-face at the MEC, while the second interview, aimed at collecting recalls for at least two non-consecutive days, took place via telephone within a period of 3 to 10 days. Daily totals of energy and 64 nutrients/food components from all food items and beverages were calculated using the Food and Nutrient Database provided by the U.S. Department of Agriculture. The intakes of vitamin A, vitamin C, vitamin E, zinc, magnesium, and selenium were compared to its respective recommended daily intake (RDI) for US adults as outlined by the Dietary Guidelines for Americans 2015–2020 (https://health.gov/sites/default/files/2019-09/2015-2020_Dietary_Guidelines.pdf). The DAQS of 0 was defined as the intake < 2/3 of the RDI and the DAQS of 1 was defined as the intake ≥ 2/3 of the RDI, respectively. The summed DAQS ranged from 0 (indicating poor quality) to 6 (indicating high quality) [18]. In our study the DAQS was classified into the two groups: < 3 group and ≥ 3 group [19].

Hypertension was the outcome in this study. For participants aged 6–13 years, elevated blood pressure was defined as ≥ 90^th^ percentile to < 95^th^ percentile or 120/80 mmHg to < 95^th^ percentile (whichever is lower) [20]. Stage 1 hypertension was defined as ≥ 95^th^ percentile to < 95^th^ percentile + 12 mmHg or 130/80 to 139/89 mmHg (whichever is lower). Stage 2 hypertension was defined as ≥ 95^th^ percentile + 12 mmHg or 140/90 mmHg (whichever is lower). For participants aged ≥ 13 years, elevated blood pressure was defined as 120/ < 80 to 129/ < 80 mmHg. Stage 1 hypertension was defined as 130/80 to 139/89 mmHg and stage 2 hypertension was defined as ≥ 140/90 mmHg [21].

### Statistical analysis

Continuous data were represented by Mean [standard error (SE)] and t-test was applied to compare differences between groups. Categorical data were represented by n (%) and chi-square test was used for difference comparison between groups. Univariate logistic regression analysis was used to explore possible confounding factors. Multivariate logistic regression analysis was used to explore the associations of overweight/obesity and DAQS with the risk of hypertension. Model 1 was the unadjusted crude model. Model 2 adjusted for age, gender, PIR, and does anyone smoke in the home, and Model 3 adjusted for age, gender, PIR, does anyone smoke in the home and energy. The combined effect of overweight/obesity and DAQS on the risk of hypertension was evaluated, and subgroup analysis was performed according to age and gender, and sensitivity analysis was performed on the data before and after interpolation of the missing values. Odds ratio (OR) and 95% confidence interval (CI) were used as effect size. SAS 9.4 (SAS Institute Inc., Cary, NC, USA) was applied to analyze the data.

## Results

### Comparisons of characteristics between participants with and without hypertension

As exhibited in Table 1, The mean age (13.82 years vs 12.79 years), and BMI-for-age (24.31 kg/m^2^ vs 20.97 kg/m^2^) of the hypertension group was higher than the non-hypertension group. The mean PIR in the hypertension group was lower than the non-hypertension group (2.44 vs 2.58). The percentage of children with BMI-for-age ≥ 85^th^ percentile in the hypertension group was higher than the non-hypertension group (51.44% vs 35.60%). The percentages of participants in the DAQS < 3 group and DAQS ≥ 3 group were statistically different between the hypertension group and the non-hypertension group.

**Table 1:** The characteristics of subjects with hypertension or not

Variables	Total (*n* = 14316)	Non-hypertension group (*n* = 12430)	Hypertension group (*n* = 1886)	Statistics	*P*
Age, years, Mean (S.E)	12.91 (0.04)	12.79 (0.04)	13.82 (0.11)	t = -9.84	< 0.001
Gender, n (%)				χ^2^ = 84.876	< 0.001
Male	7134 (50.27)	5899 (48.25)	1235 (65.13)		
Female	7182 (49.73)	6531 (51.75)	651 (34.87)		
Race, n (%)				χ^2^ = 18.886	< 0.001
Mexican American	4075 (13.05)	3534 (12.95)	541 (13.80)		
Other Hispanic	994 (6.59)	888 (6.69)	106 (5.81)		
Non-Hispanic White	4054 (58.95)	3586 (59.45)	468 (55.26)		
Non-Hispanic Black	3903 (13.23)	3271 (12.68)	632 (17.25)		
Other Race—Including Multi-Racial	1290 (8.18)	1151 (8.22)	139 (7.88)		
PIR, Mean (S.E)	2.57 (0.04)	2.58 (0.04)	2.44 (0.05)	t = 2.71	0.007
Does anyone smoke in the home, n (%)				χ^2^ = 18.349	< 0.001
No	2658 (23.11)	2393 (23.93)	265 (17.05)		
Yes	11,658 (76.89)	10,037 (76.07)	1621 (82.95)		
Weight at birth, pounds, n(%)				χ^2^ = 105.524	< 0.001
< 5.5	1285 (7.98)	1108 (7.88)	177 (8.75)		
5.5–9	7784 (56.40)	6977 (58.02)	807 (44.54)		
≥ 9	880 (6.75)	798 (7.08)	82 (4.34)		
Unknown	4367 (28.86)	3547 (27.03)	820 (42.37)		
BMI-for-age, kg/m^2^, Mean (S.E)	21.37 (0.07)	20.97 (0.07)	24.31 (0.21)	t = -16.33	< 0.001
Overweigh/obesity, n (%)				χ^2^ = 60.374	< 0.001
No	8652 (62.48)	7807 (64.40)	845 (48.56)		
Yes	5664 (37.52)	4623 (35.60)	1041 (51.44)		
Physical activity, day, n (%)				χ^2^ = 36.534	< 0.001
≥ 5	3974 (29.61)	3600 (30.56)	374 (22.61)		
3–5	748 (5.99)	676 (6.20)	72 (4.39)		
≤ 3	8427 (56.93)	7151 (55.82)	1276 (65.06)		
Unknown	1167 (7.48)	1003 (7.41)	164 (7.94)		
Vitamin A, mcg, Mean (S.E)	648.05 (7.66)	645.53 (8.36)	666.61 (16.56)	t = -1.15	0.253
Vitamin C, mg, Mean (S.E)	80.51 (1.07)	79.70 (1.10)	86.46 (3.17)	t = -2.06	0.041
Vitamin E, mg, Mean (S.E)	7.93 (0.10)	7.89 (0.10)	8.21 (0.18)	t = -1.76	0.080
Zinc, mg, Mean (S.E)	11.68 (0.08)	11.52 (0.08)	12.84 (0.21)	t = -6.20	< 0.001
Magnesium, mg, Mean (S.E)	255.16 (1.42)	252.50 (1.45)	274.79 (3.36)	t = -6.61	< 0.001
Selenium, mcg, Mean (S.E)	107.16 (0.63)	105.76 (0.66)	117.43 (1.82)	t = -6.22	< 0.001
Energy, kcal, Mean (S.E)	2248.27 (8.12)	2215.42 (8.16)	2490.08 (24.91)	t = -10.75	< 0.001
DAQS, Mean (S.E)	4.14 (0.02)	4.14 (0.02)	4.10 (0.04)	t = 0.87	0.387
DAQS, n (%)				χ^2^ = 0.442	0.506
< 3	2242 (15.71)	1926 (15.80)	316 (15.03)		
≥ 3	12074 (84.29)	10504 (84.20)	1570 (84.97)		

*S. E* Standard error, *t* t-test, *χ*^*2*^ Chi-square test, *PIR* Poverty income ratio, *BMI* Body mass index, *DAQS* Dietary antioxidant quality score

### The associations of BMI-for-age ≥ 85^th^ percentile and DAQS with the risk of hypertension

Variables with statistical difference between the hypertension group and non-hypertension group were considered as confounding factors, which included age, gender, race, birth weight, family income, does anyone smoke in the home, physical activity and energy. In the unadjusted model, we identified that children with BMI-for-age < 85^th^ percentile might be associated with decreased risk of hypertension (OR = 0.52, 95%CI: 0.44–0.62). After adjusting for confounding factors, non-obesity was associated with reduced risk of hypertension in children and adolescents (OR = 0.48, 95%CI: 0.41–0.57). No significant association between DAQS ≥ 3 and the risk of hypertension before and after the adjustment of confounders (*P* > 0.05) (Table 2).

**Table 2:** The associations of overweight/obesity and DAQS with the risk of hypertension

Variables	Model 1		Model 2		Model 3	
	OR (95%CI)	*P*	OR (95%CI)	*P*	OR (95%CI)	*P*
Overweight/obesity						
Yes	Ref		Ref		Ref	
No	0.52 (0.44–0.62)	< 0.001	0.47 (0.40–0.56)	< 0.001	0.48 (0.41–0.57)	< 0.001
DAQS						
< 3	Ref		Ref		Ref	
≥ 3	1.03 (0.84–1.27)	0.772	1.21 (0.98–1.50)	0.076	1.03 (0.81–1.30)	0.815

Model 1: Unadjusted univariate logistic regression model

Model 2: Multivariable logistic regression adjusting for age, gender, race, birth weight, PIR, does anyone smoke in the home and physical activity

Model 3: Multivariable logistic regression adjusting for age, gender, race, birth weight, PIR, does anyone smoke in the home, physical activity and energy

*Ref* Reference, *OR* Odds ratio, *CI* Confidence interval, *BMI* Body mass index, *DAQS* Dietary antioxidant quality score

### The combined effects of BMI-for-age ≥ 85^th^ percentile and DAQS with the risk of hypertension

Compared with children with BMI-for-age ≥ 85^th^ percentile and DAQS < 3, subjects BMI-for-age < 85^th^ percentile and DAQS < 3 was associated with decreased risk of hypertension (OR = 0.53, 95%CI: 0.35–0.79). People with BMI-for-age < 85^th^ percentile and DAQS ≥ 3 was correlated with decreased risk of hypertension (OR = 0.52, 95%CI: 0.36–0.74) (Table 3). Subgroup analysis revealed that in subjects aged ≥ 12 years, decreased risk of hypertension was observed in BMI-for-age < 85^th^ percentile and DAQS < 3 group (OR = 0.48, 95%CI: 0.31–0.73) as well as BMI-for-age < 85^th^ percentile and DAQS ≥ 3 group (OR = 0.47, 95%CI: 0.32–0.67) (Table 4). In boys, BMI-for-age < 85^th^ percentile and DAQS < 3 group (OR = 0.45, 95%CI: 0.25–0.81) as well as BMI-for-age < 85^th^ percentile and DAQS ≥ 3 group (OR = 0.40, 95%CI: 0.25–0.65) were correlated with decreased risk of hypertension (Table 5).

**Table 3:** The combined effect of overweight/obesity and DAQS with the risk of hypertension

Variables	Model 1		Model 2	
	OR (95%CI)	*P*	OR (95%CI)	*P*
Overweight/obesity & DAQS < 3	Ref		Ref	
Overweight/obesity & DAQS ≥ 3	1.03 (0.72–1.49)	0.857	1.09 (0.77–1.54)	0.618
Non-overweight/obesity & DAQS < 3	0.56 (0.36–0.85)	0.007	0.53 (0.35–0.79)	0.002
Non-overweight/obesity & DAQS ≥ 3	0.53 (0.37–0.77)	< 0.001	0.52 (0.36–0.74)	< 0.001

Model 1: Unadjusted univariate logistic regression model

Model 2: Multivariable logistic regression adjusting for age, gender, race, birth weight, PIR, does anyone smoke in the home, physical activity and energy

*Ref* Reference, *OR* Odds ratio, *CI* Confidence interval, *DAQS* Dietary antioxidant quality score, *RERI* Relative excess risk of interaction, *API* Attributable proportion of interaction

**Table 4:** The combined effects of overweight/obesity and DAQS with the risk of hypertension in different age groups

Variables	Age ≥ 12		Age < 12	
	OR (95%CI)	*P*	OR (95%CI)	*P*
Overweight/obesity & DAQS < 3	Ref		Ref	
Overweight/obesity & DAQS ≥ 3	1.07 (0.73–1.58)	0.715	1.10 (0.58–2.09)	0.758
Non-overweight/obesity & DAQS < 3	0.48 (0.31–0.73)	< 0.001	0.98 (0.40–2.42)	0.965
Non-overweight/obesity & DAQS ≥ 3	0.47 (0.32–0.67)	< 0.001	0.66 (0.33–1.32)	0.240

Multivariable logistic regression adjusting for gender, race, birth weight, PIR, does anyone smoke in the home, physical activity and energy

*Ref* Reference, *OR* Odds ratio, *CI* Confidence interval, *DAQS* Dietary antioxidant quality score, *RERI* Relative excess risk of interaction, *API* Attributable proportion of interaction

**Table 5:** The combined effects of overweight/obesity and DAQS with the risk of hypertension in different gender groups

Variables	Boys		Girls	
	OR (95%CI)	*P*	OR (95%CI)	*P*
Overweight/obesity & DAQS < 3	Ref		Ref	
Overweight/obesity & DAQS ≥ 3	0.81 (0.51–1.28)	0.371	1.58 (0.98–2.55)	0.059
Non-overweight/obesity & DAQS < 3	0.45 (0.25–0.81)	0.009	0.61 (0.33–1.14)	0.123
Non-overweight/obesity & DAQS ≥ 3	0.40 (0.25–0.65)	< 0.001	0.70 (0.43–1.14)	0.150

Multivariable logistic regression adjusting for gender, race, birth weight, PIR, does anyone smoke in the home, physical activity and energy

*Ref* Reference, *OR* Odds ratio, *CI* Confidence interval, *DAQS* Dietary antioxidant quality score, *RERI* Relative excess risk of interaction, *API* Attributable proportion of interaction

## Discussion

In the current study, the data of 14,316 children and adolescents from the NAHNES were collected to evaluate the influence and combined effects of overweight/obesity and DAQS on the risk of hypertension in children and adolescents. The results showed that BMI-for-age of ≤ 85^th^ percentile was associated with reduced risk of hypertension in children and adolescents, which might provide a reference for developing more targeted strategies for prevention and treatment of hypertension in children and adolescents.

Previously, various evidence indicated that children with overweight or obesity had higher risk of hypertension [22, 23]. A previous study indicated that the prevalence of hypertension was higher in individuals with excess weight and with an increased waist-to-height circumference ratio [24]. Meena et al. found that obese children had a significantly high prevalence of hypertension than normal-weight children (29% vs 7%) [25]. Another study indicated that compared with normal-weight children, obese children was associated with a higher risk of hypertension (OR = 9, 95%CI: 5.84, 13.88) [26]. Santiago et al. revealed that obese children were 2.82 times more likely to have high blood pressure than non-obese children [27]. Jakab et al. showed that the prevalence of high blood pressure was 8.3% among overweight subjects, and it was 26.7% in the obese group [28]. Obese children were more likely to develop hypertension than non-obese children with adjusted OR of 2.77 [29]. These findings gave support to the results of our study, which revealed that children and adolescents with BMI-for-age < 85^th^ percentile was associated with decreased risk of hypertension. The potential mechanism for the association between overweight or obesity and hypertension might be endocrine determinants, such as corticosteroids and adipokines, sympathetic nervous system activity, disturbed sodium homeostasis, as well as oxidative stress, inflammation and endothelial dysfunction [30]. Another study indicated that in obese people, disturbed body composition with increased visceral fat deposition, accelerated biological maturation, metabolic abnormalities typical for metabolic syndrome, and increased adrenergic drive constitutes the intermediary phenotype of primary hypertension [31]. For children and adolescents with normal weight, early interventions such as changing the poor lifestyles, doing more exercises combining with improving the diet quality was recommended.

Dietary supplements are extensively acknowledged to offer the potential to improve health if appropriately targeted to those in need, and inadequate nutrition and micronutrient deficiencies are prevalent conditions that adversely affect global health [32]. In previous studies, there was evidence showed that micronutrient supplementation was associated with reduced risk of cardiovascular diseases [33]. Zhang et al. identified that there was a reverse J-shaped association between dietary vitamin E intake and new-onset hypertension [34]. In this study, we did not identify the association between DAQS and the risk of hypertension in children and adolescents, but we found that overweight/obese and DAQS had combined effects on the risk of hypertension in children and adolescents. Dietary antioxidant nutrients such as vitamin A, vitamin E, vitamin C, zinc were reported to reduce the formation of reactive oxygen species and improve the body’s antioxidant capacity [35]. The DAQS summarizes certain dietary antioxidants and assigns a measured quantity score relative to the FDA recommended quantity, which was developed to determine the overall effect of antioxidants on health outcomes [36]. Former studies indicated that the association between dietary antioxidant nutrients and body composition related indexes may be different in children and adolescents with different body weight status. Dietary antioxidant level was negatively associated with the BMI and total fat in obese children [15]. Lower intakes of dietary antioxidant levels including vitamin C and vitamin A were associated with overweight or obesity in early adulthood [37]. In children and adolescents who are not overweight and obese, dietary approaches especially high quality diet might help decrease the risk of hypertension [38]. Regular blood pressure monitor is also important for early identifying these patients and providing timely interventions.

The current study assessed the influence and combined effects of overweight or obesity and DAQS on the risk of hypertension in children and adolescents. Compared with one kind of nutrient, DAQS included vitamin A, vitamin E, vitamin C, zinc, selenium and magnesium, which can more comprehensively reflect dietary antioxidant levels. Several limitations exist in this study. Firstly, this was a cross-sectional study, only the associations of overweight/obesity and DAQS on the risk of hypertension in children and adolescents could be found, and causal associations could not be inferred. Secondly, some possible confounding factors such as parental hypertension and overweight/obesity were not collected in the database. Thirdly, dietary intake and other data were collected through questionnaire survey, which might result in recall bias. In the future, more well-designed studies were required to verify the results in our study.

## Conclusions

This study explored the influence and combined effects of overweight/obesity and DAQS on the risk of hypertension in children and adolescents. The results found that overweight/obesity and DAQS had combined effects on the risk of hypertension in children and adolescents. The findings suggested that children and adolescents with normal weight are recommended to keep weight, do more exercise, and eat healthier diets.

## Data Availability

The datasets generated and/or analyzed during the current study are available in the NHANES database, 1999–2000: https://wwwn.cdc.gov/nchs/nhanes/continuousnhanes/default.aspx?BeginYear=1999, 2001–2002: https://wwwn.cdc.gov/nchs/nhanes/continuousnhanes/default.aspx?BeginYear=2001, 2003–2004: https://wwwn.cdc.gov/nchs/nhanes/continuousnhanes/default.aspx?BeginYear=2003, 2005–2006: https://wwwn.cdc.gov/nchs/nhanes/continuousnhanes/default.aspx?BeginYear=2005, 2007–2008: https://wwwn.cdc.gov/nchs/nhanes/continuousnhanes/default.aspx?BeginYear=2007, 2009–2010: https://wwwn.cdc.gov/nchs/nhanes/continuousnhanes/default.aspx?BeginYear=2009, 2011–2012: https://wwwn.cdc.gov/nchs/nhanes/continuousnhanes/default.aspx?BeginYear=2011, 2013–2014: https://wwwn.cdc.gov/nchs/nhanes/continuousnhanes/default.aspx?BeginYear=2013, 2015–2016: https://wwwn.cdc.gov/nchs/nhanes/continuousnhanes/default.aspx?BeginYear=2015, 2017–2018: https://wwwn.cdc.gov/nchs/nhanes/continuousnhanes/default.aspx?BeginYear=2017.

## Abbreviations

DAQS: Dietary antioxidant quality score
BMI: Body mass index
NHANES: National Health and Nutrition Examination Survey
NCHS: National Center for Health Statistics
SBP: Systolic blood pressure
DBP: Diastolic blood pressure
PIR: Poverty income ratio
RDI: Daily recommended intake
CIs: Confidence intervals
SE: Standard error
